# Book Reviews

**Published:** 1811-12

**Authors:** 


### CRITICAL ANALYSIS

OF

### RECENT PUBLICATIONS

IN THE

### DIFFERENT BRANCHES OF PHYSIC, SURGERY, AND MEDICAL PHILOSOPHY.

---

*A Treatise on Gout; containing the Opinions of the most celebrated Ancient and Modern Physicians on that Disease, and Observations on the Eau Medicinale. By* John Ring, Member of the Royal College of Surgeons in London, and of the Medical Societies of London and Paris. 8vo. pp. 208. Callow. 1811.

In the composition of this book, Mr. Ring has displayed considerable ingenuity and industry. He does not pretend to throw any new light upon this disease, to present any new theory, nor enforce any new mode of practice. His researches, however, convince us, that much may be effected towards the cure of the complaint; and demonstrate the advantage of inquiring into the opinions and practice of our predecessors decessors in the healing art. From neglecting the study of medical writers, we are apt to consider as novelties, facts which are coeval with the father of physic himself; and authors have claimed originality, when they have only had the merit of repeating what they had read.

Mr. Ring, therefore, in our estimation, has performed an useful task in briefly stating the opinions of the most famed writers, ancient and modern, on the subject of Gout. From which statement it appears manifest, that, though many of the crude notions respecting the nature and cause of the complaint have been abandoned, the mode of treating it has undergone little improvement. Indeed, were we fairly to balance the question, we think, the stimulating plan of some, and the repellent system of others among the moderns, would so much overweigh the worst practice of the ancients which could be adduced, that we should be compelled to yield them the palm. It is also pleasing to remark, how nearly the most respectable and eminent physicians, of whatever age or school, coincide in their view of treatment and cure.

Hippocrates advised purging, drinking whey and asses milk, and copious affusion of cold water to the parts affected.

"Celsus recommends bleeding immediately on the attack of the gout, and says it sometimes removes the fit for a year, and sometimes for life He also recommends temperance, frictions, warm and cold affusions, and occasional evacuations. When the swelling and inflammation are considerable, he recommends cold bathing, but gives a caution against keeping the limb in water long."

Galen prescribed bleeding, purging, and repellents. Cœlius Aurelianus clysters, cupping, or leeches; and after the fit, bathing, exercise, abstinence. Oribasius directed bleeding, purging, and friction with salt and oil at the decline of the disorder.

Ætius also prescribed bleeding and purging, cold applications, if the parts were too warm, and warm applications if they were too cold. Alexander Trallian relied on purging medicines, and in some cases venæsection. "He recommends a slender and cooling diet of every kind; and tepid water as a beverage; and tells us, he has known many people continue free from the gout, merely in consequence of their abstaining from wine."

Paulus Ægineta "recommends cooling and anodyne applications externally; a cooling and diluting diet; bleeding and purging at the attack of the disorder, and exercise and friction at its decline. He then advises the patient to live abstemiously, and, if possible, to refrain altogether from wine; adding that he has known a number of persons, all of whom have thus recovered their health."

Of the moderns, Mr. Ring commences with Mayerne, who recommends occasional bleeding, vomiting, and purging, in the attacks and intervals of the gout; and observes that water-drinkers are seldom troubled with this complaint; and advises those who cannot totally refrain from wine, to drink it only in moderation, and much diluted.

Willis says, "bleeding is often of service in a recent case of gout, or one that is not of long standing, especially in a warm constitution, and at the commencement of the disorder, otherwise it does more harm than good."—"He thinks emetics proper in those constitutions where they agree, where they operate with ease, and are safe on other occasions."—"He also recommends abstinence, and alteratives, particularly a milk diet; but remarks, it does not agree with every constitution." He also advises anodynes as well as evacuations. Sydenham, who suffered much in his own person from gout, did not appear very decided in his practice, or sanguine in expectation of the disease being radically cured, though he thought a remedy might hereafter be discovered. He allows that bleeding, purging, and sweating, seem to be indicated in the gout, yet thinks we should not so far encroach on the prerogative of nature, but suffer her to eliminate the peccant matter in her own way, by insensible perspiration. He recommends temperance and exercise.—Cornaro, Cheyne, Cadogan, and Darwin, cured themselves of gout by abstinence; and, with many other authorities, may be cited in support of the disease being curable. Baglivi, Hoffman, Boerhaave, Van Swieten, Mead, Cadogan, Heberden, and others amongst the moderns, also may be quoted in favour of bleeding, purging, and abstinence in gout.

Mr. Ring has successfully combated some opinions of Dr. Latham, published in a Treatise on Rheumatism and Gout, in which he contends that these disorders are not of an inflammatory nature.

Dr. Kinglake's Dissertation on Gout also is sharply attacked, and poor Dr. Brown is "awaked from the dead," to be brought before the tribunal of our author, who has not even spared his domestic misfortunes.

The latter pages of the book contain some observations on the Eau Medicinale; and a copious history of its introduction and success in this country, forming altogether a good, though rather severe commentary on Dr. Jones's Treatise on that nostrum, the value of which seems to be duly appreciated by Mr. Ring.

We shall conclude with an extract from his Remarks on the the Cure of Gout, which seem to result from considerable experience, and merit attention.

"The gout is an inflammatory disease, and, like other inflammatory diseases, can only be cured by the cooling or antiphlogistic plan; yet as it seldom or never occurs but in habits previously debilitated, either by indolence, intemperance, vehement mental affections, or unusual exposure to cold, it requires great judgment and extensive knowledge of the healing art, to carry this plan into execution.

"In plethoric habits, and in others when the symptomatic fever is violent, bleeding in the arm is necessary. Local bleeding is necessary in some cases, and advisable in all. This is best done by leeches; but in some instances, where leeches could not be procured, great relief has been derived from opening a vein in the vicinity of the part affected, or from simple scarification.

"Emetics should either not be prescribed in a paroxysm of the gout, or prescribed with great caution. They are particularly dangerous when there is any sign of fulness of blood, or an inflammation of the stomach, which is indicated by sickness, with pain in that organ.

"Cathartics are in general useful in the gout, and have furnished the principal materials of the regular and empirical remedies, for that and other inflammatory disorders in all ages. They should never be omitted when constipation prevails; nor when the heat of the skin, and hardness of the pulse, indicate that the phlogistic diathesis runs high. Sudorifics also are sometimes of service in arthritic, as well as in rheumatic affections. They should not be selected from the stimulant, aromatic kind, nor given in a large dose, to bring on a profuse sweat, but administered in such a manner as only to promote a gentle perspiration.

"The best emetics are the preparations of antimony and ipecacuanha; the best cathartics are, manna, salts, rhubarb, magnesia, calomel, scammony, and senna. The best sudorifics are, antimonials, or ipecacuanha, in small doses, volatile salines, tepid diluting liquors, and temperate warmth.

"Opiates, though endowed with an anodyne and sudorific property, should never be given till after the remission of the inflammatory symptoms, unless in conjunction with a cathartic.

"Strict attention must be paid to regimen in the gout; especially during the paroxysm of the disorder. The patient should then abstain from all sorts of animal food, aromatics, and fermented liquors, and live on arrow-root, water-gruel, panada, sago, or other farinaceous food. He should drink some mild diluting beverage, such as barley-water, toast and water, or tea.

"During the increase and height of the fit, he should keep his bed, or at least remain at rest, and in a recumbent posture; but when a crisis has taken place, and pain, and other symptoms of inflammation abate, advantage may be derived from a slight motion of the joint, or from gentle friction, which may be gradually increased.

"Even when the patient is in a state of convalescence, and his appetite keen, abstinence, or at least temperance, should be enjoined, and he ought to be very cautious how he indulges himself in the luxuries of the table, otherwise a relapse may take place. Bitters, and other provoca-

 tives tives of the appetite, should either be totally interdicted, or cautiously prescribed. The best tonic, in general, in such cases, is moderate exercise; which, if regularly performed, steadily persevered in, and accompanied with temperance, will seldom fail to restore health, and invigorate the system."

---

### *Journal Generale de Medecine, de Chirurgie, de Pharmacie, &c.* No. 174, Fevrier, 1811.

( ontinued from Page 405.)

*Sur les differens modes de traiter le tetanos en Amerique, &c. Par le* Dr. Louis Valentin.—In North America, the usual mode of treating tetanos consists in topical applications, stimulants, opium, mercury, and the cold bath.

Dr. Rush, during the American war, was convinced from great experience, that opium was inefficacious in tetanos, on account of its sedative and debilitating properties. He therefore substituted for opium, musk, and other antispasmodics, wine, bark, in large doses, and blisters along the spine. When he perceived that the wine and bark began to lose their action, he added oil of amber*.

In a subsequent memoir, he appears to place greater confidence in opium, if given in considerable doses. When a person has the first indications of tetanos, the Professor recommends an emetic, a strong dose of laudanum, the warm bath and cinchona. If there is a wound, it is to be laid open, and inflammation and suppuration to be excited. By these means he has succeeded more than an hundred times in preventing the disease. He has also seen good effects produced by cold baths and cold affusion, and in some cases advises the patient to be wrapped in wet sheets.

Many cases are recorded in various journals in which mercury proved beneficial; but it is extremely difficult to obtain the mercurial action in sufficient time.

In some instances benefit was derived from the use of polygala senega, in others of tincture of cantharides, and in some of tobacco glysters.

*Trismus nascentium* is very common and very fatal among the negroes in the West Indies. Dr. Valentin has never seen a case cured.

This Dissertation upon the whole displays considerable research and knowledge of the disease, but the result affords us

---

* Transactions of the American Philosophical Society, vol. 2, and Medical Inquiries and Observations, by B. Rush, M. D. vol. 1.

little little satisfaction; seldom as tetanos occurs in this country, our means of cure are at least as successful as those employed in America, where, in the cities of Philadelphia and New York from six to nine individuals annually die from the complaint. In the West Indies it is yet more frequent.

---

*Extrait de l'Histoire d'un Somnambulisme, &c. Par* M. Dessessartz.—The subject of this case when a child was extremely delicate; the period of his infancy was passed in sickness, and he was reared with difficulty. Between three and four years of age he had a low nervous fever; at eight and a half, hooping cough, which was severe and obstinate. At nine and a half he was attacked with putrid fever, which continued fifteen days. Six months afterwards, in the beginning of autumn, he complained of violent pains in the head, not continual or regularly periodic, but very frequent. Various remedies, chiefly anti-scorbutics, were prescribed, and the symptoms yielded. Three weeks afterwards they recurred, and at the commencement of the autumn became insupportable. Vapour baths were ordered, and continued once or twice a day for three months. The cure appeared radical till the autumn, when the child again experienced pain of the head less violent, but attended with a sense of weight; these again yielded to the vapour-bath.

In the summer of 1805, being in his fourteenth year, he entered as a cadet in the navy at Petersburgh. He embarked and continued on board ship two months without experiencing any inconvenience.

In the month of October he was attacked with colic, and continued vomiting, not only of alimentary but of glareous matter; he was not intemperate, and did not suffer from sickness whilst at sea.

In the night of the 18th—19th of September, 1806, three weeks after returning from a cruize at sea, during which he had suffered no inconvenience, commenced the somnambulism and other affections about to be described.

The patient was found in an insensible state at a distance from his chamber. Blisters were applied. On recovering his senses, he had no recollection of having left his bed or his chamber. He swooned twice in twenty-four hours, his mouth being wide-open, and his limbs stiff as if dead; the pulse continued beating. At intervals he was seized with trembling and signs of fear. Sometimes during the paroxysm he got out of bed, and if he had not been supported would have fallen down. The disease increased daily. Occasionally the paroxysms were characterized by an appearance of choaking or or strangling, from which the patient relieved himself by thrusting his fingers as far as the base of the tongue, and provoking vomiting of glareous matter; at other times, he was affected with terrible convulsions, alternating with fright and fury without any evident cause, striking and biting himself.

At this period a physician was called in and medicines were employed. On the 17th of October, the 30th day of the disease, he was suddenly deprived of the use of his legs, after a slight paroxysm which had only continued a quarter of an hour, though the former ones had been longer. For eighteen days he was obliged to use crutches, except during the paroxysms, when the legs resumed their power and participated in the violent action of the whole body.

The fourth of November, the seventeenth day of being in this state, the patient could not support himself and was obliged to remain in bed; this weakness continued three days, when he resumed his crutches, and seven days afterwards the legs regained their power and motion. As this alteration took place the paroxysms put on the tertian type; and their duration was habitually from an hour to an hour and a half.

*Beginning of the Somnambulism.*—Elixir of vitriol had been substituted for the most powerful anti-spasmodics, bark, and vermifuges; and in eight days the trembling, signs of fright, choakings, stranglings, paroxysms of rage, in which the patient struck and bit, were subdued, and a calm somnambulism was established.

During a short interval when the young man possessed his reason, he was suddenly surprized with a trembling in his hands, and a sleep which scarcely endured a minute and a half. Immediately afterwards he employed himself in mathematics, and reading some books of amusement. These symptoms recurred at the decline of every paroxysm till the termination of the disease.

The patient continued in this state, with some slight variations, from the 26th of November, 1806, till the 12th of March, 1808.

The author of the memoir has arranged the different appearances of the somnambulism under sixteen periods, of these we shall select those which are interesting and explanatory of the disease.

### *Type and variation in the Course of the Disease.*

From its commencement the disease manifested itself by paroxysms which continued every day an hour or an hour and a half. The 26th of November of the same year, 1806, the paroxysm, roxysm recurred every second day, between eight and nine o'clock in the evening, and continued in this type till the 16th of March, 1807. At this epoch it varied : sometimes two paroxysms occurring in one day, one in the morning, another in the evening. This continued till the 7th of April, during which time, there were only two days with one paroxysm ; but whether double or single, they preserved the tertian intermittent character.

These symptoms continued with greater or less urgency, with longer or shorter intervals of relief, till March, 1808, when they entirely ceased. Between the paroxysms, when they took the type of tertian intermittent, the young man was in full possession of his faculties, studied mathematics, history, and belles lettres, with close attention, and diverted himself with conversation and amusements agreeable to his age.

He was fond of tea and coffee, preferred beef to any other meat, disliked wine and liquors, and was gentle in his manners.

During the paroxysm, if, which was frequently the case, he was occupied in mathematical labours, his operations were rapid and accurate. The logarithmal calculations in which he was engaged demanded strict attention, and a long series of combinations ; hence he had daily to resolve new problems for the first time, which could not be attributed to a mechanical reproduction of memory. Before he inserted the solution in his book, he tried it on a slate, and if he found it incorrect, began it over again. When he had copied this solution, he collected his papers, instruments, &c. and placed them in order. It seemed as if the symptoms, which usually terminated the paroxysm, waited for this moment, for they immediately evinced themselves and the fit ended.

If the paroxysm occurred whilst he was reading, he resumed his book after the fit, and continued the subject where he had left off, though he had not marked the place. He expressed his wishes by signs or in writing, and sometimes he spoke, and even kept up a continued and rational conversation ; thus after having read a portion of the Iliad, he conversed upon it by question and answer.

In all circumstances his eyes were open, and his look was natural ; he saw objects but did not always recognize them. Thus, having received a letter from his father who promised to come to him in the spring, he stood musing for half an hour at a window which looked into the court-yard, without making any reply to the questions which were addressed to him. He then ran hastily into his chamber, took his bed-furniture, coverlets, verlets, sheets, matrass, and pillows, arranged them in the saloon; placed the table beside the bed, and covered it with every thing necessary for a repast, which he got from the buffet. Hitherto the organ of sight appeared to be faithful; but though his nurse was close by him, and a relation who had never quitted him, he complained that from the moment his father was expected, every body had gone out, and that he was left alone. He returned to the window, reperused his father's letter, looked for some time stedfastly on the court-yard, and withdrew to his chamber, whither he was followed by his relation and the nurse. He remained a little while without seeming to observe them, at length he recognized his relation, and thanked him for returning so *a propos*. Some time afterwards he saw his nurse, who was at a little distance, and embraced her to express how glad he was to see her come back again to the house. He returned to the casement, and feeling that the paroxysm was near its termination, threw himself on the bed. Convulsive shaking of the hands and feet, and a short sleep came on, and he presently arose, asked the hour, and ordered tea to be brought.

The organ of hearing did not appear to be affected; only when reading low, being requested to read louder that he might be heard; he remained stupified, and had a violent convulsion: when this was over he asked for paper; some was presented him on which was written the request to read louder. He no sooner perceived the word *louder* than he was attacked with fresh convulsions. Upon offering him another piece of paper, he wrote quietly.

The sense of touch did not suffer. The digestive organs underwent no alteration. His appetite indeed was small, but digestion was well performed, and the bowels were open, but the stools were slimy. The pulse was usually at 80 or 82.

In some paroxysms he appeared terror-struck, in some he was very furious; and occasionally he was in a state of stupor, motionless, his limbs stiff. Almost always he complained of acute general pains, more severe in the legs and head than in other parts; these he had experienced for several years.

The complaint was considered by the physicians as decidedly nervous, complicated according to some of them with worms, because the patient had voided some during the fever when he was nine years and a half old. One practitioner in high repute, not only considered the affection as nervous, but alleged that it depended upon a scrofulous habit.

In consequence of these opinions, the most powerful antispasmodics, bitters, tonics, vermifuges, and vapour-baths were were prescribed; and they several times afforded so much relief that the cure was thought complete.

Since the autumn of 1807, the fits having become more violent, and the above-mentioned remedies proving ineffectual, in February, 1808, an English physician (Dr. Leyghton) was called in.

Upon examining the patient very minutely, he felt a considerable beating in the epigastric region, and pronounced that the chief seat of the disorder was in the organs of digestion, to which the remedies must be directed, and affirmed that when these were restored to a healthy condition, the nervous affection would cease. The former stimulating remedies were in consequence discontinued; he adopted the purgative plan which had been proscribed, and with this view directed a table-spoonful of castor-oil to be taken every evening. Just as the patient commenced this treatment, he was seized with great debility, which obliged him to use crutches or a stick for three days; he was not, however, disconcerted, but said he felt as if he should not have a fit. This was the case, the fit, which had regularly occurred every day for three months and a half, missed that day, but returned on the following day, and became tertian. The paroxysms continued about four hours, violent on the onset and termination, but milder in the middle, though severe pains in the head were experienced throughout. The castor-oil, which was regularly given every evening for twenty days, occasioned in about half an hour, a copious evacuation of fœces of very bad appearance, some of them being hard, and long retained; others more recent, but ill digested, the urine was thick. These circumstances induced the physician to visit his patient during a paroxysm. On the 25th of February, three days after having commenced the remedy, Dr. L. accordingly saw the young gentleman towards the end of a fit. He spoke little, knew what he said, and recognized the persons around him, and his signs indicated severe pains in the head. Dr. L. continued the castor-oil, and directed some pills with aloes, calomel, soap, and ginger; three of them, and afterwards four were taken an hour before supper, and the oil was given when going to bed.

A strict regimen was enjoined, in which were excluded, coffee, beans, peas, spinach, &c. and purslain which was always found badly digested. The treatment being continued, the digestive power improved, the beating, which had been felt in the epigastrium, gradually diminished, and the pulse became less nervous. At length, in March, 1808, the fit which was expected at nine in the morning did not occur, and has not returned since. The medicines, however, were continued as usual for six days longer, when the quantity was diminished; minished, the pills were discontinued altogether on the 19th of April; but an aperient mixture was still taken to keep the bowels open. After forty days of convalescence, he was allowed to go on board ship, with proper precautions respecting his diet.

---

*A Paper, containing the Results of eleven years Practice at the Original Vaccine Institution, No. 44, Broad-street, Golden-square, &c. &c. &c.* Written by the Medical Board of the Institution. 8vo. Lond. 1811. pp. 46.

In a former number of this Journal we gave an extract from this Pamphlet, sufficiently indicative of its character, and containing, as we conceived, results of considerable interest, deduced from extensive experience, and calculated to illustrate the natural history, and prophylactic properties of Vaccina. As the result of observations, apparently given without partiality or prejudice, we cannot but recommend it to the notice of the Profession. The following "*Directions for the Vaccine Inoculation*," we think may be generally useful.

"1. The limpid matter should be taken from a decidedly characterized cow-pock, which is proceeding, apparently, through its respective stages. It is most efficacious in producing the vaccina from a pock before the eighth or ninth day; but is most abundant, and is usually taken, about the ninth day.

It may be used at any earlier period, even as early as the fifth day, if it can be collected. However, matter from a pock later than the eleventh or twelfth days is not more liable to produce inflamed arms than that from younger pocks; and if the cow-pock be excited at all, it is as distinct as from any earlier matter. No differences in the effects of the vaccine matter inoculated appear to depend on the presence, extent, or absence of the red areola.

"2. The matter is usually taken on glass, thread, or a quill, on which it should be suffered to become dry without applying heat; and when so dried it is scarce visible. The matter may be kept fluid between two glass plates, in one of which a small cavity has been drilled, or in a bottle filled with hydrogen gas.

"3. To produce more effectually the unsusceptibility intended; and as dried matter fails much more frequently to excite the vaccina than recent fluid matter, it will be adviseable, that, instead of a single puncture or scratch, there be matter inserted in two or even three punctured or scratched parts in each arm. If the constitution be affected, one pock is as effectual in producing the unsusceptibility required, as any greater number; but the chance of the constitution being affected, seems to be greater from several, than from a single pock.

pock. The dried matter at the time of inoculation should be softened by warm, but not very hot water.

"4. The inoculation must be performed in the same manner as for the small-pox.

"5. If the infectious matter produce the required effect in three, four, or five days, there will be seen a red spot like a small gnat bite—in six or seven days, a small vesicle will appear—in nine days, a circular vesicle (improperly called a pustule) will be found as large as a pea, or from about two-tenths to four-tenths of an inch diameter, usually surrounded by a red areola.—By the eleventh day, the vesicle begins to scab or grow dry, and turn black in the middle, and the areola becomes more extensive.—By the fifteenth day, but often later, the pock becomes a mere scab, circular, prominent, well defined, of a blackish or mahogany colour, adhering firmly; but the areola disappears. Unless it be separated by violence, the scab does not fall off, in general, sooner than the twentieth day. It then leaves a cicatrix permanent for life.

"6. If the eruption or pimple, excited by inoculation, has not the characters and does not pass through the stages in the course above stated (5), although sometimes anomalous, this cow-pock may render the constitution unsusceptible of the small-pox, yet it cannot be depended upon. In such cases the inoculation should be re-instituted; for if the vaccina cannot be again excited, the unsusceptibility desired will have been produced; but if a further proof be wanted, recourse must be had to inoculation with the variolous matter.

"7. In many cases, no constitutional affection or fever can be perceived: when it occurs, it is almost always on the ninth and tenth days; but provided the pock exhibit the distinctive characters of the cow-pock, even without areola, with the usual course of its stages, the susceptibility of the small-pox will be generally as effectually destroyed, as if there had been considerable febrile affection, and an extensive areola.

"8. Experience having at length shewn that persons who have gone through the vaccina with all the known appearances of the most effectual sort, are susceptible of the small-pox, although in the proportion of at the most, one out of 500, it is advisable to re-inoculate either cow-pock or variolous matter in a few months after the first inoculation.

"9. If erythema, like erysipelas, extend over the arm with swelling, pain, &c. it has always subsided in a few days of itself, only avoiding irritating applications, or at most on using sedatives.

"10. Eruptions sometimes occur, but they require no particular treatment.

"11. The small-pox may break out at any period within twelve days of inoculation for the cow-pock. If they appear earlier than the sixth or seventh, the vaccina is cut off in its progress; if they appear later, the vaccine-pock goes forward in its usual course.

"12. The medical treatment which may be required from un-

 usual usual or supervening complaints, is similar to that in the small-pox.

"13. Measles, chicken-pox, hooping-cough, and other disorders may intervene during the vaccina, without, in general, varying its progress.

---

### *The Edinburgh Medical and Surgical Journal*, No. XXVII.

*Case of Asthma, cured by Stramonium*.—Continued from Page 244.—Mr. English, a medical practitioner, here relates his own case. In very early life, when a boy, he was subject to wheazing and shortness of breath upon any slight cold. When in China, in 1799, he was seized with a severe paroxysm of spasmodic asthma, accompanied with hepatitis. The paroxysms of asthma have since had frequent returns.

"The paroxysm commences with the usual symptoms of cold, together with purging and much evacuation of urine, succeeded by intolerable flatus in the stomach and bowels, frequent convulsive cough, constant wheezing, with painful dyspnœa, being unable to fill the chest with air: any sudden exertion, speaking above a word or two together, or attempting to walk up a hill or up stairs bring on suffocation; much frothy tenacious *saliva* is discharged from the throat, with some congealed phlegm from the bronchia, and in the mornings, what is expectorated is often streaked with blood, and sometimes a little pure blood is coughed up. The paroxysm runs its course in from three to five days, when the flatus subsides, and expectoration becomes free and easy; being, instead of frothy or tenacious *saliva*, and the jelly or white of egg-like substance, common phlegm, with a little good looking pus."

Here is jumbled together a collection of very formidable symptoms, and had we not been assured, by persons who seem to have the means of knowing, we should doubt their being related by a person educated in the medical profession. Who would expect to hear a surgeon say that "instead of frothy tenacious *saliva*, and the jelly or white of egg-like substance, common phlegm, with a little good looking pus," were expectorated. Notwithstanding we find *catharsis*, *dyspnœa*, *submurias hydrarg*. &c, &c. in this production, we still doubt its being written by a surgeon, because we have never met with a person actually of that profession, who could say that "frothy tenacious *saliva*" was at any time expectorated from the bronchia. The unadulterated herb was smoked; the cure is stated to have been rapid, and permanent. As we have no doubt of the power of Datura *stramonium* over certain cases or states of difficult respiration, we are concerned to see any equivocal histories of its effects laid before the public.

*Notes on Diabetes Mellitus, as it occurs in Ceylon.* By THOMAS CHRISTIE, M. D.—THESE notes were written at Columbo in Ceylon, in September, 1809: they contain a detail of several cases of diabetes, all of which were either relieved or cured by pursuing the method suggested by Dr. Rollo. The disease subsided in proportion to the strictness with which the patient adhered to the use of animal food.*

It appears, from these notes, that *diabetes mellitus* is of more frequent occurrence in Ceylon, than on the continent of India, or in Europe. The fact of the frequent occurrence of diabetes among the Cingalese is endeavoured to be accounted for from the diet of these people.

The bulk of the people, in the vicinity of Columbo, consume, I believe, less animal food than in most parts of India, and certainly far less grain, which, from the nature of the soil, and the state of cultivation round Columbo, is not produced in nearly sufficient quantity for the subsistence of the people. A considerable quantity of rice is imported from Bengal, and other parts of India, but the poorer orders have seldom the means of procuring much rice, and live a great deal upon yams and sweet potatoes, jack-fruit, plantains, or bananas, and particularly cocoa-nuts, which form a great part of the subsistence of the people in the corles, or districts, dependant on Columbo. These all furnish sugar ready formed, and in greater abundance than rice, besides which they use a great deal of country sugar or jaggery, prepared from the toddy of the rittiel palm (caryota urens), which is very cheap, and forms an article of export from this place.

"As a strong corroboration of my opinion, that it is owing to the immoderate use of these articles, that the Cingalese are so subject to diabetes, it ought to be mentioned, that during nine years, in which I have received the hospital returns of all the European and native troops on Ceylon, not a single case of diabetes has occurred amongst them, although the number of men has in general been about 7000, and that of sick has been on an average upwards of 500. The Europeans have beef served out to them daily, and the natives receive a liberal allowance of rice, on which they chiefly live.

"If it should be allowed, that the natives of Ceylon are more exposed to attacks of diabetes in consequence of their using little animal food, and subsisting chiefly on articles furnishing a great deal of sugar, it will readily be understood, on the general principle, that the system is acted on most powerfully by those causes to which it is

* There is a remarkable fact in the history of medicine connected with one of these patients, Don Juan Appoo, a Cingalese physician. The practice of this person used to be principally among small-pox patients, but of which he had not seen a case for the last three years, and is now obliged to keep a school as a means of subsistence. This strongly corroborates the accounts of the extirpation of the small-pox in the island of Ceylon, by the introduction of vaccination.

least least accustomed; that the abstraction of all articles of diet which contain sugar, or from which it is likely to be formed, with the substitution of an animal regimen, will be more likely to produce an immediate change on the state of the urine and digestive powers, than with Europeans who have been accustomed to a more considerable proportion of animal food in their ordinary diet.

"Few of the people of Ceylon, who are plain and simple in their general mode of life, are ever disposed to indulge much in the luxury of the table; so that unless it interferes with their religious principles, or with the state of their circumstances, a change of diet is with them a subject of little consideration; and on that account I have found less difficulty in prevailing on them to comply strictly with my injunctions, which in general went to the entire prohibition of every thing but animal food of different kinds, dressed with ghee, or clarified butter, and water, with or without the addition of a little brandy.

"It ought to be remarked, that in all my patients there was an increased appetite. Had they been in that advanced stage of diabetes in which the appetite fails, it might have been dangerous to abstract so suddenly all their accustomed articles of food, and advisable only to adopt the animal regimen gradually or partially."

The following extract, translated from the *Yoga Ratnakere*, the name of a collection of prescriptions in the Cingalese language, will, at least, gratify curiosity. The *Pra mehé*, or diseased flow of urine, is divided into twenty species, of which ten proceed from phlegm, and are easily cured; six from bile, which may also be cured with care: and four from wind, which last are incurable. In this language diabetes mellitus is called *madu mehé*, honey urine.

"The ten species proceeding from phlegm are,

"1*st*, Udaka mehé.—*Udaka* in Sanscrit and Pali signifies water, and the symptoms are a flow of very clear urine, cold, without smell, like water, and discharged without pain, with a little slime.

"2*d*, Ikshu mehé.—*Ikshu* Sanscrit and *utchu* Pali, means the juice of the sugar cane, and the symptoms are slimy cold urine, like that juice.

"3*d*, Sura mehé.—*Sura* in Sanscrit and Pali signifies toddy, and in this the urine resembles toddy, and on being kept deposits a similar sediment.

"4*th*, Sandra mehé.—*Sandra* in Sanscrit signifies thickness, and in it the urine, after being allowed to stand a night, is thick.

"5*th*, Pishta mehé.—*Pishta* Sanscrit, *pitha* Pali, signifies flour, and in this species the urine, after standing a night, deposits a sediment white as flour, and of very little weight. Another symptom is that, when the urine is passed, the hair of the body stands on end from pain.

"6*th*. Sukra mehé.—*Sukra* Sanscrit, *suka* Pali, signifies semen, and in this the urine is discharged with a mixture of semen, which it resembles in colour.

"7*th*, Saikta mehé.—*Saikta* Sanscrit, *sikata* Pali, signifies sand, and in this this species small round particles of phlegm are discharged with the urine, resembling sand.

" 8*th*.—Sita mehé.—*Sita* in Sanscrit, Pali, and Cingalese, signifies cold, and the symptoms of this species are extremely cold and very sweet urine.

" 9*th*, Samairima mehé.—*Samairima* in Sanscrit signifies drop by drop, and the symptom is the urine being passed drop by drop.

" 10*th*, Alala mehé.—*Alala* Sanscrit, *lala* Pali, signifies saliva, and the symptoms are urine mixed with particles of phlegm, in the shape of small threads, and slimy like saliva.

" The six species proceeding from bile are,

" 1*st*, Manjesta mehé.—*Manjesta* Sanscrit, *manjutta* Pali, is the name of a red seed, and the urine in this species is of a reddish colour, and has a fishy smell.

" 2*d*, Rakta mehé.—*Rackta* Sanscrit, *ratha* Pali, signifies blood, and the urine in this species is of the colour of blood, feels hot, tastes salt, and has a fishy smell.

" 3*d*, Nila mehé.—*Nila* in Sanscrit and Pali, signifies blue, and in this species the urine is of a bluish colour.

" 4*th*, Hariddra mehé.—*Hariddry* Sanscrit, *halliddy* Pali, signifies yellow, and in this species the urine is of a yellow colour, of a sharp taste, and discharged with heat.

" 5*th*, Rala mehé.—*Rala* in Sanscrit and Pali signifies black, and the urine in this species is of a sooty colour.

" 6*th*, Kshara mehé.—*Kshara* Sanscrit, *kara* Pali, signifies saltish, and in this species the urine is to the smell, taste, and touch, like sea-water.

" The four species proceeding from wind are,

" 1*st*, Wasa mehé.—*Wasa* in Sanscrit and Pali signifies fat, and in this species the urine is discharged frequently, either mixed with fat, or entirely composed of an unctuous substance.

" 2*d*, Mudja mehé.—*Mudja* Sanscrit, *minja* Pali, signifies marrow, and in this species the urine is discharged frequently, and resembles marrow, or is mixed with that substance.

" 3*d*, Hasta mehé.—*Hasta* Sanscrit, *hutty* Pali, signifies elephant. The urine in this species is discharged frequently, and with difficulty, resembling the semen of a ruttish elephant, or the liquor of the joints.

" 4*th*, Madu mehé.—*Madu* in Sanscrit and Pali, signifies honey, and in this last species the urine is of the colour and taste of honey.

" In the *Bayajja Manjussy*, or Medicine Chest, another work more lately translated from the Pali, the same account is given of the different species of *mehé*, and it is said they are occasioned, amongst other causes, by whatever produces much fat, urine or phlegm, as indolence, eating cold and sweet things, and food of a watery nature. The more immediate causes of the *madu mehé*, are either decrease of the substance of the body, and excess of wind, or the wind's being obstructed and mixed with the blood, on which account it is incurable. The distinguishing symptoms of the four species of *mehé* proceeding from wind, are flatulence and eructation, tightness of the chest, tremor, pains, restlessness, emaciation, and difficulty of breathing.

" The

"The only remedy recommended for *madu mehé*, is pills composed of seventeen ingredients, amongst which are the following: Sulphur, nitre, borax, sal ammoniac, yellow arsenic, cinnabar, capsicum, black pepper, and several other vegetables with which I am unacquainted.

"Although the above division and distinctions of the *pra mehé*, which seems to include all the diseases of the urine, is arbitrary, and often fanciful; yet it is a curious circumstance, that the Indian physicians should have described so distinctly the sweetness of the urine in *madu mehé*, which had escaped the observation of both the ancient and modern physicians of Europe till the time of Willis."

---

*Case of Tic Douloureux, cured by Arsenic; by* Mr. M'Kechnie, Surgeon.—This case of Tic Douloureux was in a man 57 years of age, of a healthy constitution. Various methods were resorted to for the removal of this painful affection. Blistering, mercury to salivation, cicuta, topical bleeding by leeches, were all found ineffectual. In the attack which occasioned the relator to be consulted, the pains were most troublesome by day, seldom disturbing the patient in the night.

"Excruciating paroxysms were excited by the slightest irritation; as by a breath of cold air blowing upon the face; by attempting to chew, or by speaking abruptly, and sometimes even without provocation. At these times the pain suddenly darted into the lower jaw, and from that into the cheek, and beneath the ear. Each exacerbation consisted of a great number of short paroxysms, which lasted about a minute, then remitted, returning immediately, again remitted, and so alternately, generally for about the space of an hour. The exacerbations were not constant in degree or duration. They sometimes caused violent contortions of the face, most ease being found by twisting the mouth to the left side, so as to stretch the muscles of the affected places, and by rubbing the skin towards his mouth with his fingers; relief was sometimes procured by pressing firmly over the infra-maxillary foramen. When the anguish was greatest, the face flushed, and tears gushed from the eyes, but no change in the appearance of the parts affected could be discovered. The patient described the pain to be at one time, as if something was piercing or screwing into the flesh; at another, as if it was tearing or twisting from the bone. When the paroxysms ceased, he had perfect mmunity from pain, till the morbid action was again excited. The general health was not impaired; the pulse was of the usual standard; the appetite was good; the bowels were regular."

Under the above circumstances the patient took the *Solut. Arsen.*——As the maximum dose of the remedy was approached to, the symptoms gradually subsided, and at length entirely subsided, and a permanent cure was effected.

*Case of Tumours of the Tongue cured by Mercury.*—A woman 47 years of age, the mother of a numerous family, and still menstruating regularly, but whose health had suffered fered from abortions and floodings, about two years and a half before the time here spoken of, was attacked with great pain in the tongue, which swelled to such a magnitude that she could not speak, or swallow any thing but fluids. This swelling subsided in six weeks, but left several small knots in the substance of the tongue, round the edge of its anterior portion. These tumours were irregular in their surface, having various depressions and elevations, and were as hard and unyielding as scirrhus. They were all painful; and the sensation they gave was as if a spear was thrust from them into the inferior jaw. By the use of the blue pill, and frictions, so as to produce considerable ptyalism, these tumours were completely removed.

*Case of Recovery from an excessive dose of Laudanum.*—A woman 28 years of age swallowed an ounce of T. opii. The symptoms were alarming, but by the use of emetics and cathartics they were removed; and the patient recovered.

*Observations upon Herpes of the Prepuce.*—The disease, of which some cases are here given with remarks, was first noticed in this Journal, (vid. Med. and Phys. Journal, Vol. 23. p. 441, No. 136, for June, 1810), where a history of its appearances and progress was given, with a plate shewing the changes from day to day. Before the account there given, we believe no writer had mentioned this complaint; neither does it appear, prior to that publication, to have been understood by practitioners, but was often treated for syphilitic chancre, with consequences hazardous, injurious, and sometimes fatal. In the paper in the Med. and Phys. Journ. above cited, it was denominated an "*Acute Eruptive Disease of the Integuments of the Penis*;" a term which seems clearly to characterize its appearance, symptoms, and progress. About a year after the publication of this paper in the Med. and Phys. Journ. a writer who is a reporter from the Carey-Street Dispensary for the Edinburgh Journal, noticed this disease under the term *Herpes Preputialis*. When speaking of it, he observes, "I know of no writer who has mentioned the herpes of the prepuce, except one, in a Number of the Med. and Phys. Journal for June, 1810, *who has given a plate very ill representing its form.*" As we admit the reporter to be a person of considerable research in medical literature and the history of diseases, we consider the first part of the sentence above quoted very strongly to establish the claims of the writer of the paper on an "*Acute eruptive Disease on the Integuments of the Penis*," in the Med. and Phys. Journal for June, 1810, to priority of description. That part of the sentence which roundly asserts, that the

 plate plate annexed to the paper in the Med. and Phys. Journal, but ill represents the form of this disease, we must be allowed to say, probably was suggested by the reporter's never having seen the disease in the form which it appeared to the writer of the paper. Perhaps he had then only seen the disease as it occurs within the fold of the prepuce, and where both the vesicles and subsequent ulcers are placed in contact with the glans, as the prepuce is brought forward. Under that circumstance of locality, the plate does but ill represent the form of the disease; neither was it ever intended to represent the disease so placed. We are the more confirmed that the reporter has taken his idea of the disease from cases where the vesicles have been placed within the fold of the prepuce, by his speaking of its going through its stages in fourteen days; whereas, when the vesicles are placed on the tegument of the body of the penis, a very frequent occurrence, it goes through its stages, and is completely well, in ten days at farthest. It was this last form of it from which the drawing was taken; and a great number of instances, since that period, have satisfied the writer of the paper in the Med. and Phys. Journal, that his representation is a faithful transcript from nature. When the disease is placed on that part of the tegument of the penis, which certainly is not prepuce, and never comes in contact with the glans, the most severe cases we have yet seen, if not irritated by the "busy hand of art," have been completely healed in ten days. When the disease has been placed within the fold of the prepuce, we have never yet seen it get well in less than fourteen days; and we have seen the ulcerations continue for five or six weeks. Beside being protracted in its duration, the disease when placed upon the inner fold of the prepuce is much altered in its appearance: the vesicles are less distinct and pellucid: the ulcerative process is more irregular, and never has the hardened crust which is represented in the drawing, and described in the history, as indicating its last stage. We are disposed to believe that the true and distinctive character of the disease is to be taken when it is neither disturbed by art, nor interrupted in its progress by the accidents of situation. It would have been wrong to have taken the character of the variolous pustule from those found about the fauces: it is wrong to take the character of this species of *Zoster* from vesicles placed within the fold of the prepuce, because from that accident they are altered in appearance, progress, and duration. We are little inclined to verbal disputes, or we might object to the term *Herpes preputialis*, because the disease is considerably turned from its natural course when placed within the prepuce; and because, as far as our observation servation has gone, and a great number of cases have fallen within our practice, we have seen it five times in seven situated on a part of the integument of the penis which is not prepuce.

The cases before us add something to the history of this disease, though our experience has not taught us that it is to be repelled or shortened in its duration by art, as the writer of these observations asserts. We believe that the interference of the surgeon may prolong but not shorten its duration: we know of no instance in which it has been repelled, nor are we in the least aware of any prophylactic process which should prevent its recurrence; for when it has once happened it returns again and again, at intervals of a few weeks. The profession is indebted to Mr. M'Kechnie for again bringing the subject forward, and inducing the surgeon to attend to the history of a disease which has confused and misled the most experienced, and which by being misunderstood, has occasioned serious mischief to many individuals.

---

*Case of Diabetes treated by Blood-letting. By* Mr. S. M'Keur, Surgeon.—We transcribe the minutes of this case chiefly with a view to shew the curious fact as before asserted by Mr. Watt, of the blood getting a firmer texture, and assuming the inflammatory appearance, as bleeding was persisted in.

"*November* 16*th*.—Pulse 100, so feeble as to be with difficulty numbered; much oppression in the chest. ℥viii. of blood were taken from his arm, being all that could be procured; veins so relaxed and empty that it is with difficulty any of them can be opened; pulse not altered by the bleeding, but feels a good deal relieved in the chest; laxative pills *pro re nata*.

"17*th*.—Pulse scarcely to be felt; veins cannot be made tense by ligature; ℥viii. of blood again procured with much difficulty: the energy of the heart seems almost gone; no alteration from the bleeding; the blood taken yesterday covered with a thin blue film; the substance of it very loose, and black in the bottom; serum exceedingly white; a large blister to be applied over both kidneys.

"18*th*.—Blister rose well, but discharges little; other symptoms as before. Urine about xxlb. in the 24 hours; ℥x. of blood again taken; no sensible effect from it; blood taken yesterday of the same appearance as before.

"19*th*.—Pulse 104, more regular, and somewhat fuller; blister *now* runs well; ℥xii. of blood taken; *now* for the first time it runs in a full stream; felt relief while flowing; pulse rather improved by bleeding.

"20*th*.—Blood taken yesterday firmer in texture than any formerly, and contracts more on the surface, with a thicker buffy covering; pulse as as yesterday; ℥xii. of blood again taken; veins now fill better, and the blood flows more forcibly.

"*21st.*—Pulse about 100, considerably firmer; ℥xvi. of blood taken; much relief while flowing; pulse not altered; lime-water ordered.

"*22d.*—Pulse 120, but quite distinct, feels very languid; palpitation very distressing; considerable oppression about the præcordia: on the whole very unwell; drink and urine considerably diminished; blood taken yesterday can be suspended on a probe; feels most comfortable in bed, and cannot walk without difficulty; stools for the last two days rather brown, and without any blood; tongue a little cleaner; mouth still very bad tasted. Ordered powders of ipecacuanha and oxyd antimon. c. phosphat. calcis.

"*23d.*—Sweated some through the night; feels to-day easier; pulse as before; ℥x. of blood again taken in a very full stream, and with great force; pulse sensibly weakened by it; otherwise it was intended to have taken a much larger quantity; much relieved while flowing; never any tendency to syncope.

"*24th.*—Thirst lessened, in other respects as before; ℥xv. of blood taken; flowed with great force as in pneumonia; pulse before bleeding 108, after it 110, and much weakened; blister to be repeated.

"*25th.*—Blister discharges freely; serum of the blood taken yesterday of the appearance of the matter of a scrofulous abscess, crassamentum has the tenacity of healthy blood; considerable buffy coat; has much less thirst, and voids about one-third less urine than before; thinks his mouth not quite so bad tasted as before; has sweated a good deal; skin softer and more natural; urine not quite so sweet.

"*26th.*—In the morning while in bed, pulse 104, after being up 120, tolerably firm; rather more thirst to day; urine not increased; ℥x. blood taken, flowed more feebly; bowels regular; stools brown; sleeps tolerably.

"On the 28th, ℥xii. of blood taken; on the 29th a similar quantity. No material circumstance occurred till December 1st, when a violent diarrhœa came on, with severe griping; drunk little since it came on; urine diminished to one half; pulse 100, and firm; little appetite; feels very languid; stools still brown.

"*December 2d.*—Diarrhœa continues; stools very dark brown, very languid; griping very severe; pulse 100, tolerably firm; urine for these last two days not above ii lb.; thirst also greatly diminished.

"*3d.*—Diarrhœa diminished; urine increased to the same quantity as before the diarrhœa, but drinks less; pulse as yesterday. Pills of calomel and ipecacuanha ordered.

"*4th.*—℥xii. of blood taken; pulse weakened by it; in other respects as before.

"*5th.*—Blood taken yesterday tolerably firm; ℥xii. more taken; pulse weakened again by it.

By this process the diabetic symptoms were relieved, but the patient did not recover his health.

*Facts*

*Facts and Observations on Burns. By* Mr. LYALL.—Mr. Lyall, while House-Surgeon to the Manchester Infirmary, gave a particular attention to the treatment of burns. His observations have led him to approve the stimulating applications as advised by Dr. Kentish, to which he adds the exhibition of stimuli internally. When the thorax or abdomen are the seat of the burn, peritoneal, &c. inflammation follow the use of cold applications it is asserted: therefore, says Mr. Lyall, "When burns, by whatever cause produced, or to whatever extent, happen on the thorax or abdomen, I would never use cold dressings, but have recourse to the warm terebinthinate applications, or, if I wished a milder dressing, to the Carnon oil. *Linim. Aq. Calcis.*"

---

*Observations on the Use of the Eau Medicinale, and of Rhubarb, in the cure of Gout. By* G. BURROUGHS, Esq.—As the delirium of expectation raised in the public mind by the histories of cases of Gout cured by the miraculous Eau Medicinale has not quite subsided, it remains a duty to state every authentic fact respecting its operation and effects: with this impression on our minds, we give the two cases published by Mr. Burroughs of Clifton.

"Sir E. H. an Irish baronet at this place, aged about 70 years, was the first person that I had seen taking it. He had been troubled with gout at various periods during thirty years, of which disease distorted joints bore the most ample testimony; independently, however, of these appearances, his body exhibited no mark of disorder, and for his time of life might be considered healthy and strong. The attack of gout in which he began with the *eau medicinale* was exceedingly severe, and pretty general, attacking ancles, knees and elbows. In this state, with much avidity he took half a bottle of this medicine; but not finding any amendment from this dose, after an interval of six hours, he had recourse to the remaining portion. This, however, soon began to have effect, and operated most powerfully by all the secretions, occasioning at the same time violent vomiting, purging, sweating, and acting no less violently by the kidnies. These powerful effects continued without intermission for at least forty-eight hours, when the patient became exhausted to the very last extremity, and at the time my interference was requested, appeared almost lifeless; body motionless, and covered with a cold moisture; voice nearly inaudible, and the powers of perception scarcely remaining. Nothing for some time could appear more unpromising. By diligent and unremitting attention, however, with the aid of warm Madeira and cordials, the exhausted powers were supported, and at length raised; but the patient long remained in a state of considerable weakness, and his convalescence was for some time doubtful. It is unnecessary for me to say, that this fit of gout was completely removed by this violent process,—a cure which almost cost the patient his life. In one particular, perhaps, the patient erred, and that was by taking the second dose too soon after the first. The period between each each may be thought should have been longer than six hours; but it should be remembered, that the pamphlet published on its merits states, that some persons take a whole bottle at a time;—how then are the public to discriminate when they should take a larger or a smaller quantity? The baronet certainly had some prudence in the matter; for he did not take the remaining half bottle, until a lapse of six hours had given him every reason to suppose that it would produce no effect. Had he unfortunately taken the contents of a whole phial in the first instance, there is every reason to suppose that he never would have recovered the shock. I have stated so much to shew, that the medicine is very powerful, and that great care and attention is necessary in the use of it, and that patients disposed to try it should, in the beginning, measure their way very carefully."

Mr. Barry, a respectable watchmaker at Clifton, here relates his own case.

"About the middle of January, 1811, I was threatened with symptoms which generally portend *a regular* fit of gout; its violence, however, did not, as on most preceding occasions, increase in any particular part, so as to deprive me of rest, or cause actual confinement, till about the 23d, when the attacks in the stomach becoming much more frequent and formidable, I determined on taking the celebrated eau medicinale, two bottles of which I had long kept by me for that purpose. I began with half a bottle, which for two hours seemed to produce little or no effect. I then felt considerable pain in the head, whether of real gout, or the effect of the medicine, I cannot say: this was soon succeeded by delirium, afterwards a great nausea in the stomach, purging, and sickness, but not the smallest disposition to perspire. This continued eight or ten hours, when I began to expect the wished-for relief, of which I had so long and so confidently flattered myself the medicine would afford me; but, alas! I found the disorder increasing, both as to extent and virulence, and, after a lapse of four or five days, I took the last half bottle, with, however, no other effect than a nausea of much less duration than the former, still, however, without the wished-for success. Being then apprehensive that I had not taken a sufficient dose, I resolved, after a similar interval, to take the remaining whole bottle at once, which had much the same effect in every particular as the first half bottle, with this difference only, that the sickness was of much longer continuance, but not sufficiently violent to enable me to vomit without great difficulty. The nausea on the stomach remained for several days, and the gout there, as well as in the extremities, evidently gaining upon me, presented no other appearance or feeling, than that I must shortly sink under it. Much about this time there appeared all over my body an eruption resembling very much the measles; but as I had had that disorder many years before, I concluded it must have been occasioned by the medicine. A few days after this I was seized in bed very suddenly with a copious spitting of blood, attended with a wheezing from the throat, and coughing, which for a while threatened suffocation; this, it is supposed, must have arisen from the rupture of some vessel about the lungs, by efforts made during the sickness to vomit.

"The hæmorrhage continued to return in an alarming degree, at intervals, intervals, for a week or ten days, till, weakened by its frequent recurrence, and apprehensive, if longer neglected, it would inevitably lead to the most dangerous consequences, I called in two medical gentlemen to my assistance, by whose skill and attention I was soon restored to a state of rapid recovery.'—Here is a very clear and able description of a violent fit of gout, in which the *eau medicinale* had a very fair trial, and wherein it not only completely failed, but was soon followed by a considerable aggravation of the complaint, and with symptoms threatening the most imminent danger,—delirium, violent vomiting, intense pain, the body covered with eruptions, and accompanied with feelings and appearances as if he had been poisoned; to these succeeded an alarming bleeding from the lungs, placing his life for many days in a state of imminent danger. The patient had often had attacks of gout that were fully as violent as this in the commencement, but were never followed by such distressing and alarming symptoms as appeared on this occasion; it is therefore but reasonable to suppose, that the very unusual disturbance in his system was occasioned by the remedy. How far to refer the hæmorrhage from the lungs to it, may perhaps appear difficult, unless we suppose there may have been some translation of gout to that part, as well as to the stomach and head; or perhaps some vessel may have been ruptured in the lungs from the violent straining in vomiting. Whatever was the cause, the effect was productive of great danger and alarm."

---

*Practical Observations on Cancer: by the late* JOHN HOWARD, Surgeon Extraordinary to the Cancer Ward in the Middlesex Hospital. 8vo. Lond. 1811. pp. 144.

THE munificence of the late Samuel Whitbread was directed to encourage an investigation of the causes and cure of *Carcinoma;* and by his friendship with Mr. John Howard, the fund which he appropriated to this humane and laudable purpose was employed to found an Establishment in the Middlesex Hospital, the express object of which was the treatment of this deplorable malady; exclusively, however, as it appears in the female. Accordingly women only are admitted into the Cancer Ward. Mr. Howard, by being appointed Surgeon Extraordinary to this establishment, was led to a particular attention on Cancer; and the "Practical Observations" now before us are the result of that attention. On the death of Mr. Howard the papers which compose this volume were bequeathed by their author to Dr. Charles Gower, of Old Burlington-street, and to that gentleman the public is indebted for their revision and publication.

The time that has elapsed since Mr. Howard collected these observations has not superseded their publication by any successful treatment of cancer being discovered; for among the improvements, improvements of modern practice we have not the good fortune to enumerate a remedy for carcinoma.

These observations, as their title expresses, are of a practical nature, interspersed, however, with numerous desultory remarks, not unfrequently assuming the character of hypothesis. It is advantageous to the publication to remark that the first class is most numerous, and in the 63 cases which are given, there must necessarily be found many instructive facts. As a specimen of this part, we shall cite two instances, one of *Noli-me-tangere*, with its treatment, and the other, a new species of Cancer, so called by the Author.

"The *Noli-me-tangere* of the face takes its rise from a small beginning, like a pimple or little wart, which is probably a diseased miliary gland of the skin. The discharge after ulceration produces a scab or crust. Should that crust be rubbed off from time to time, it is exposed to the air, being sometimes in an incrusted state and sometimes as a sore, from picking or handling. Under these circumstances, with a strong predisposition in the habit, a creeping and spreading ulceration comes on, slowly if the part be little irritable; but it is sometimes so irritable that the mischief extends with great rapidity. An incipient case of the former kind, having the appearance of a small tumour on the side of the nose, I once cured, by keeping the part covered constantly with a powder composed of two parts of Lapis Calaminaris, finely levigated, and one part of Pulvis Cerussæ. The employment of this dry, astringent, sedative, and, I may add, incarnative powder, to painful, irritable, and phagedenic sores, was so far new as to be my first time of using it. I had, many years ago, seen the *Pulvis Fuscus* used in St. Bartholomew's Hospital, to hasten the cicatrization of sore legs. This was a composition of Lapis Calaminaris, levigated with a small quantity of myrrh, with which the sores were powdered with a puff, and covered, without lint, with Ceratum Epiloticum. It was very useful in promoting both incarnation and healing; and, if I mistake not, the same, or a similar application was employed by the antients to promote the healing of wounds and sores. Now, as Cancer resembles, very much in one respect, a phagedenic sore, for, like it, it is highly irritable, often spreads, and destroys the skin, cellular membrane, and glands, even at a great rate, I adopted this composition by analogy in the above case, as it was not only likely to remove the extreme difficulty to give ease, but to prevent the extension of corroding mischief."

Corroding ulceration is necessarily of difficult management, and under treatment apparently founded on the most rational principles, often so rapidly increases in its destructive progress, that a name expressive of this property has been given to it. It was in this peculiar case that Mr. Howard found this drying powder effect a cure. He mentions a second case, in a much older person, where the ravages vages of the disease were checked, for years, by this application. It appears that the hints in part was received for this, from a similar composition employed by Mr Adair Hawkins, who, however, did not employ it in cancer, but had frequently applied it to phagedenic bubo. His method of using it was in the form of a dry powder, but combined with Cort. Peruv. This remedy was powdered on with a puff, so as to make a wall or covering of the composition. As fast as the covering cracked the crack was filled up, to the exclusion of the external air; and, under this, the spreading phagedenic sore was healed.

The following case is singular in its form, its progress, and its result.

" A gentleman, aged about 45, was attacked by, probably, a new species of Cancer. Having, as he conceived, slept in a dirty bed abroad, a hardness and scaly eruption came on one shoulder, nearly upon the head of the humerus, resembling lepra, but which terminated in slough. This appearance, after some time, subsided, and caused the mark of a cicatrix of a previous ulceration. After the complaint had left this shoulder, in a few months it attacked the other below the joint of the shoulder, upon the upper part of the deltoid muscle, where a kind of indolent tumour, or hardness, arose, about the size of an egg; not in the muscle itself, but in the integuments. This hardness went on slowly through the course of some months, and without much pain, until the skin became of a purplish hue, like a boil, but not so painful; and this was followed by a slough, which separated in a sluggish way, and left the muscle bare, deprived of the cellular and adipose membrane, and a part of the skin. Then the sore healed. The gentleman's health was in a declining and precarious state, before the appearance of this disease, and as it went on he recovered. Finding only a local and external complaint, under increasing health, it was suffered to go on. I had never seen any thing similar, and it seemed to be a nondescript. I was happy to find that the ravages this complaint made were external, and that health was restored. It is now sixteen years since its first appearance; he has had, at least, fifty such tumours, during that time, following precisely the same natural course. As one goes on to cicatrization, others arise, and proceed in the like course, vegetating, increasing, and sloughing. The time of growth of these tumours seems to be during the winter and spring; and the time of sloughing, which gets to its height in August and September, is during the summer; after which, the sores heal. From the shoulder to the elbow, the skin is all scar; and the disease has sometimes extended to the fore-arm, having passed over the joint without producing diseases within: it has, however, occasioned some degree of contraction; and the tumours which have formed below it, pass upon the flexor muscles of the fingers. This very singular affection is probably constitutional and leprous; its seat is in in the skin, and in the cellular and adipose membrane under it. It produces ultimately a sloughing of the common integuments. It affects the subjacent parts, viz. the muscles, only in as far as the sloughing leaves them naked, and without natural covering; it neither ulcerates the skin, as a herpes, nor does it corrode the subjacent parts. It is impossible to conjecture what could have determined this sort of humour or affection to the arms only; but I am inclined to think that if the like humour had fallen upon the integuments of the scrotum, being immediately in the vicinity of an important glandular part, a cancer might have been occasioned, resembling those before described."

Books have been written upon diseases which should not be cured. Though we are equally at a loss with the author to say what could have determined this affection to the arms, yet we do not hesitate to class it with those complaints which should not be cured. It was doubtless, a mode which nature, the vis medicatrix, the archeus, or by whatever name we call that mysterious and powerful principle which exists with vitality, followed to expend the morbid actions of the frame. We have had the misfortune often to see an unexpected loss of life follow speedily on the subsidence of accustomed morbid irritations, whether arising spontaneously or effected by art; by the latter mode, however, we expect most hazard is incurred.

(*To be continued.*)

